# The Role of the Nurse in the Management of Irritable Bowel Syndrome: A Narrative Review

**DOI:** 10.37825/2239-9747.1043

**Published:** 2023-12-22

**Authors:** Luigi Ruggiero, Vincenzo Andretta, Carlo Soldaini, Ivan Fricano, Marco Mancusi, Annamaria Sellitti, Josephine Vastarella, Antonella Santonicola

**Affiliations:** a Gastrointestinal Unit, Department of Medicine, Surgery and Dentistry “Scuola Medica Salernitana”, University of Salerno, Baronissi, SA, Italy; b AOU San Giovanni di Dio e Ruggi d’Aragona, Salerno, Italy

**Keywords:** Healthcare improvement, Nursing education, Nursing leadership

## Abstract

Irritable bowel syndrome (IBS) is characterized by chronic symptoms of abdominal pain in association with changes in bowel habits. Abdominal pain is the most debilitating symptom for IBS patients, and its management is one of the greatest challenges for gastroenterologists. In recent years more evidence has arisen about an increasingly central role of the nurse in the management of gastrointestinal diseases including IBS. The aim of this narrative review is to analyse the latest evidence on the pathophysiology, diagnosis, and treatment of IBS patients with a specific focus on the role of the nurse in its management.

## 1. Introduction

Irritable bowel syndrome (IBS) is a chronic functional disorder defined according to Rome IV criteria by the presence of recurrent abdominal pain (at least 1 day per week in the past 3 months) associated with altered defecation or bowel habits [1].

IBS is part of the disorders of the brain-gut axis (DGBI), and it is estimated that more than 40 % of people worldwide meet the criteria for one of the various DBGIs [2]. The prevalence of IBS worldwide using the Rome IV Criteria is 3.8 % [3]. IBS affects 1.5 to 3 times more females than males [4,5]. It occurs in all age groups [5] with a symptom onset by age 35 years in more than 50 % of patients [6].

IBS is associated with reduced quality of life [7] and psychological comorbidities such as anxiety and depression [8]. This results in a significant economic impact on health care due to the high number of visits to gastroenterology outpatient clinics [9,10] and increased prescription of drugs [11] or inconclusive instrumental tests (e.g., colonoscopy) [12].

Among the symptoms of IBS, chronic abdominal pain represents the greatest cause of patient discomfort [13] and is therefore the greatest challenge for the physician to address. Epidemiologic data suggest that the prevalence of chronic unspecified abdominal pain is approximately 22.9 per 1000 person-years, and up to 25 % of the adult population suffers from abdominal pain at any time [14,15] without differences in age, ethnicity, and geographic regions [16,17]. The difficult management of pain in IBS and other comorbid pain conditions requires more support from the nurse who can play a key role in this context.

The aim of this narrative review is to lay out the basics of pathophysiology, diagnosis, and therapeutic approach in patients with IBS with a focus on the role of the nurse in its management.

## 2. Methods

Our study design was set up as a narrative review in order to describe to GI nurses their role in the diagnosis and management of IBS. Then, we searched for the more relevant scientific studies that could provide information about evidence-based management of IBS.

A review of the scientific literature in English through databases that included Cochrane, Pubmed, and Scopus was conducted. Studies from January 2000 through June 2023 were selected.

## 3. Pathophysiology

The International Association for the Study of Pain defines abdominal pain as “An unpleasant sensory and emotional experience associated with actual or potential tissue damage or described in terms of such damage” [18].

The pain afferent pathways start from the primary spinal afferent neuron that terminates in the dorsal column of the spinal cord; the second-order afferent neuron projects to the thalamus and midbrain, and the third-order neuron is part of a network of integration of gut sensory information, which affects the variability of pain experience and signalling [19]. In addition, nerve tracts descending from the brainstem may influence the sensitivity of dorsal horn neurons and as such may be considered a central control mechanism of pain perception [20].

IBS is considered a disorder of the brain-gut axis because the neurobiological mechanisms underlying this pathology involve both a peripheral component of pain stimulus genesis and a central alteration of afferent inputs. This explains the disparity between the intensity of chronic pain and the severity of tissue damage that leads healthcare providers to underestimate the intensity of pain compared with what patients report.

Visceral hypersensitivity represents one of the main pathophysiological mechanisms underlying IBS. Colonic distension has been shown to result in decreased pain threshold, increased intensity of sensation, and exaggerated viscerosomatic referral [21–23] with the finding of increased severity of gastrointestinal (GI) symptoms [24]; but there is no linear relationship between the objective intensity of a gut stimulus and the sensation experienced.

The Central Nervous System (CNS) also seems to play a role in the pathogenesis of IBS and the generation of symptoms. Recent functional magnetic resonance imaging studies have found altered emotional arousal, cognitive control, and endogenous modulation of pain in patients with IBS compared with controls due to different processing of the somatosensory processing regions (e.g., thalamus, insula) and limbic and paralimbic regions (e.g., amygdala) [25,26] suggesting that this results in different descending endogenous modulation of pain. Such brain responses have also been confirmed by studies during aversive rectal distension [27,28]. Altered central processing has been shown to play a more important role than visceral hypersensitivity in patients with more debilitating abdominal pain [29].

Psychological distress may also result in lowering the threshold of afferent signals to reach conscious awareness leading to greater symptom intensity [30] or may also be a risk factor for the evolution of an acute peripheral insult in the gut in the context of chronic pain [31].

In recent years, evidence has suggested that alterations in the gut microbiota (dysbiosis) may contribute to the pathogenesis of IBS. The GI tract is composed mainly of Firmicutes, Bacteroidetes, Proteobacteria, and Actinobacteria [32], and these organisms release various signalling molecules and metabolites that are critical for intestinal homeostasis and the development of the mucosal immune system [33]. Consequently, even minute changes in the composition of the gut microbiota can generate inflammatory changes that result in altered intestinal permeability and translocation of bacteria across the mucosa [33]. Numerous data have found alterations in the gut microbiota in patients with IBS although the results were conflicting and no significant differences between subtypes of IBS were found [34,35]. The three major meta-analyses found decreases in Bifidobacterium, Lactobacillus, and *Faecalibacterium prausnitzii* [36], or increased levels of Firmicutes and decreased levels of Bacteroidetes [37], or lower levels of Lactobacillus and Bifidobacterium and higher levels of *Escherichia coli* and Enterobacter [38].

In addition, it has been hypothesized that small intestinal bacterial overgrowth (SIBO) may also be correlated with the pathogenesis of IBS. A meta-analysis found that 49 % of patients with SIBO diagnosed by lactulose breath test and 19 % of patients diagnosed by glucose breath test had a diagnosis of IBS [39]. Further studies are needed to demonstrate this relationship and understand its underlying pathophysiological mechanisms.

## 4. Diagnosis

Diagnosis of IBS can be complicated since symptoms are usually very generic and may change over time [40] or mimic other disorders [41,42]. Considering the absence of a gold standard diagnostic test or biomarker for IBS and to allow standardized diagnosis, symptom-based diagnostic criteria have been developed and the most widely used are the Rome Criteria. These criteria have been modified and revised over time. They changed from the Rome III Criteria introduced in 2006 [43] to the Rome IV Criteria introduced in 2016 [44]. The new criteria were much more restrictive than the previous ones because they eliminated abdominal discomfort from the definition of IBS and increased the frequency of the abdominal pain that must occur to meet the criteria for diagnosis of IBS.

Indeed, the diagnosis of IBS now requires the presence of recurrent abdominal pain, at least 1 day per week in the last 3 months with the onset of symptoms for at least 6 months associated with two or more of the following criteria: related to defecation, associated with a change in the frequency or shape of stools [44].

A careful history is essential to rule out any warning signs that require specific instrumental and laboratory investigations such as blood in the stool, unexplained weight loss (>10 % in 3 months), nocturnal symptoms, fever, and family history of colon cancer, celiac disease, or chronic inflammatory bowel disease.

In addition, a physical examination and rectal exploration should be performed. If central sensitization is prominent, it is common to observe a larger painful area on palpation of the abdomen or other painful sensations during the investigation. Abdominal pain in a patient with symptoms consistent with IBS is not a warning sign, but its intensity should be assessed to rule out concomitant conditions underlying intense symptoms.

The diagnosis of IBS should be made at the first visit and be confirmed by normal laboratory tests such as complete blood count, c-reactive protein, and celiac serology, supplemented by faecal calprotectin when diarrhea is the predominant bowel habit [45].

Drossman et al. recommend the development of a multidimensional clinical profile that includes five dimensions of disease: diagnosis based on Rome IV, any subclassification relevant to treatment such as the predominance of abdominal pain, impact of the disease on patient-defined daily life, relevant psychosocial modifiers, and physiological modifiers if clinically relevant, e.g., a colonic transit test [46].

IBS can be further divided into four subtypes based on the Bristol Stool Form Scale (BSFS), which characterizes stool consistency from hard to soft on a scale of 1–7 [47,48]:

- IBS with predominant constipation (IBS–C): More than one quarter (25 %) of bowel movements with Bristol type 1 or 2 stool form and less than one quarter (25 %) of bowel movements with Bristol type 6 or 7 stool form.
- IBS with predominant diarrhea (IBS-D): more than one-quarter (25 %) of bowel movements with Bristol type 6 or 7 stool shape and less than one-quarter (25 %) of bowel movements with Bristol type 1 or 2 stool shape.
- IBS with mixed bowel habits (IBS-M): more than a quarter (25 %) of bowel movements with Bristol type 1 or 2 stool shape and more than a quarter (25 %) of bowel movements with Bristol type 6 or 7 stool shape.
- Unclassified IBS (IBS–U): Patients who meet the diagnostic criteria for IBS but whose bowel habits cannot be accurately classified into one of the three groups above should be classified as having unclassified IBS.

## 5. Treatment

### 5.1. Basic treatment

A key role is played by doctor-patient communication in which the patient should be reassured that the symptoms are not life-threatening. Advice on lifestyle and physical activity should also be given [49].

### 5.2. Dietary advice

Dietary advice is one of the first questions asked by the patient during the medical examination. Foods such as dairy products, those containing incompletely absorbed carbohydrates, spicy and fatty foods, foods containing wheat, and those associated with histamine release have been shown to be associated with worsening GI symptoms in patients with IBS [50].

One of the most recommended diets is the low FODMAP diet. FODMAP stands for fermentable oligosaccharides, disaccharides, monosaccharides, and polyols, which are short-chain carbohydrates (sugars) that the small intestine absorbs poorly, leading to impaired water reabsorption and gas production for fermentation by the gut microbiota in the colon. The low FODMAP diet involves a three-step process that should be guided by a dietitian, in which initially all FODMAP content is eliminated for four to six weeks and then individual groups of FODMAPs are gradually reintroduced to identify dose-dependent symptom triggers, ending with a phase in which the diet is individualized [51].

Little scientific evidence supports traditional dietary advice drawn up based on the guidelines of the National Institute for Health and Care Excellence (NICE) and the British Dietetic Association. Such diets are based on recommending healthy eating habits (adequate fluid intake, regular meals, not eating too much or too little) and to reducing intake of foods that could cause symptoms (e.g., spicy, fatty, and processed foods; alcohol, caffeine, and carbonated beverages; commonly consumed gas- and fiber-producing foods; limit intake of fresh fruit to a maximum of three per day) [52]. The NICE diet is recommended as the first choice in the recent guidelines [49,53].

The gluten-free diet showed mixed results probably because only a portion of patients with IBS-D respond to that diet [54–56].

All three diets improve IBS symptoms, but the low FODMAP diet is the most evidenced-based dietary intervention in reducing abdominal pain in patients with IBS [57,58]. However, in a recent head-to-head study among the three diets in patients with non-constipated IBS, most patients preferred the advice of the traditional diet, which was much easier to follow [59]. The low FODMAP diet and the gluten-free diet can be chosen as second-line treatments according to the patient’s preferences, but it should be guided by dieticians.

Probiotics as a group are effective in reducing overall symptoms of irritable bowel syndrome and abdominal pain [60], without strong recommendations on specific species or strains. If probiotics are tried, it is recommended to use them for a longer period, such as 12 weeks, before deciding on their effectiveness.

### 5.3. Peripheral neuromodulators

Peripheral neuromodulators include antispastics that exhibit anti-muscarinic properties or are composed of peppermint oil (L-menthol), which acts as a κ-opioid receptor agonist and 5-hydroxytryptamine 3 (5-HT3) receptor antagonist. Antispasmodics are generally used for the treatment of intermittent abdominal pain. Recent evidence has shown their superiority in abdominal pain in IBS over placebo [61,62].

Guanylate cyclase C receptor agonists act by increasing intracellular cyclic guanosine monophosphate (cGMP) levels in intestinal mucosal cells with effects on submucosal nociceptive neurons. These drugs cover primarily IBS-C resulting in increased mucosal fluid excretion with consequent acceleration of intestinal transit. Recent evidence on Linaclotide and Plecanatide has shown a positive long-term effect in treating the abdominal pain component in IBS-C [63,64].

Serotonin receptor agonists/antagonists (5-HT3 and 5-HT4 receptors) are mainly effective on intestinal transit (5-HT3 receptor antagonists slow it down and 5-HT4 receptor agonists accelerate it), but they also seem to have some effects on abdominal pain. Studies have been conducted on Alosetron [65], Ramosetron [66], Ondansetron [67], and Prucalopride [68], but they are currently not routinely used in the treatment of IBS because of poor evidence or side effects.

### 5.4. Central neuromodulators

Treatment with central neuromodulators is the second line of treatment in patients unresponsive to general measures, diet, or peripheral neuromodulators. In patients who respond to treatment, there is a lack of evidence on when to reduce or stop treatment. To date, expert consensus recommends continuing treatment for at least 6–12 months, and longer in individuals prone to symptomatic relapse [20].

Tricyclic antidepressants (TCAs) are the first-line treatment for IBS when abdominal pain is dominant. They act by inhibiting the reuptake of 5-HT and norepinephrine and have an antagonistic action on 5-HT2A and 2C, muscarinic, and histamine receptors. Side effects may include dry mouth, constipation, and sedation because of proarrhythmic potential, a baseline electrocardiogram is required, and use is not recommended in individuals who have had a myocardial infarction. Numerous evidence has shown a reduction in abdominal pain in patients with IBS with treatment with tricyclic antidepressants at a dosage of 25–50 mg/day [20,69].

Norepinephrine serotonin reuptake inhibitors were recommended by a recent Rome Foundation Working Team review as second-line treatment of abdominal pain in IBS when TCAs are not tolerated or have an insufficient effect [20], although official evidence is lacking. Serotonin and norepinephrine reuptake inhibitors have the advantage of having relatively few side effects compared with tricyclic antidepressants, even when constipation or depression in comorbidity is part of the disease. The most common side effect is nausea, which can be reduced if taken with food and tends to decrease after the first week of treatment. Duloxetine can be used in a range of 30–90 mg/day, while venlafaxine requires a dose of at least 225 mg/day.

Selective serotonin reuptake inhibitors may be chosen when comorbid anxiety or depression is considered important to the experience of pain in IBS, although there is no formal evidence of a pain-reducing effect [20].

### 5.5. Psychological therapy

Psychological treatment has been shown to be crucial in managing IBS symptoms, including abdominal pain, and improving patients’ anxiety, depression, and quality of life. It is currently recommended as a second line of treatment. Current evidence shows good results of this treatment even in the long term [69,70].

The most widely used therapy and among the most effective for abdominal pain in IBS [69,71,72] is cognitive-behavioral therapy, which is based on treating negative thoughts that affect symptom perception, anxiety, and behaviors by suggesting avoidance strategies [73,74].

Alternatively, hypnotherapy [75,76] can be used, which is based on creating a state of physical and mental relaxation in which the person is more susceptible to the suggestions used by the therapist [77]. Gut-focused hypnotherapy usually includes 6 to 12 treatment sessions over three months, in which the therapist provides gut-focused verbal suggestions and uses guided imagery to reduce attention and anxiety toward IBS symptoms and pain perception [78]. There are also improvements in global IBS symptoms, gut-specific anxiety, and quality of life with long-term effects [77].

## 6. The role of the nurse in the management of IBS patients

Nurses can play a key role in most of the above reported treatment options as shown in Table 1. For this purpose, specific knowledge is required. Heitkemper et al. selected 100 nurses who were interviewed by telephone about their knowledge and opinions on IBS. One of the most striking findings was that only 13 % were aware of the diagnostic criteria for IBS and more than 50 % stated that they would need more training in the management of IBS [79]. Therefore, there is a need for GI nurses to gain knowledge on the main pathophysiological, diagnostic, and therapeutic aspects of IBS. Moreover, intensive clinical training in Functional Bowel Disorders enables them to properly manage IBS and to recognize the presence of other intestinal or extraintestinal comorbidities associated with IBS.

One of the ways in which nurses can have the greatest positive impact on IBS patients is through their education and support. The use of nurse-led basal self-regulation strategies on the theory of self-management can improve the patient’s symptoms and their quality of life [80–82]. Recently, Chen et al. compared the results of using online education modules on self-management of pain, symptoms, and quality of life in patients with IBS alone and with nurse counselling, showing that both improved pain and quality of life; however, nurse counselling was superior in improving patients’ quality of life [83].

Regarding dietary treatment, nurses can explain to patients the important role of diet considering that most IBS patients modify their diet in an attempt to control their symptoms, without any supervision from a healthcare specialist and this could lead to nutritional deficiencies [84]. Recently, it has been demonstrated that GI nurses can even supervise specific dietary advice such as Low FODMAP or NICE diets, that are a cornerstone in IBS treatment. In fact, Sultan et al., in a recent review, suggest that dietary advice can be delivered by other health professionals such as GI nurses, after specific training [85].

Abdominal pain is the cardinal symptom reported by IBS patients and the nurse could help patients to manage their IBS complaints. Lin et al., in 2004 evaluated the effect of nurses’ preoperative intervention in abdominal pain before and after abdominal surgery, demonstrating an improvement in preoperative pain anxiety and attitude and postoperative abdominal pain [86]. Similar results have also been obtained in other studies [87–89]. Therefore, we can speculate that GI nurses could obtain similar results in IBS patients.

Finally, in specific settings and after dedicated training, nurses can take part in the psychological therapy of IBS patients. In 2004, Smith demonstrated that nurse-led hypnotherapy results in an improvement of symptoms, quality of life, and abdominal pain in IBS patients [90]. Recently, Murray et al. showed that nurses can also substitute for health care professionals in exposure-based cognitive-behavioural therapy with improvements in IBS symptoms [91].

## 7. Discussion

Our narrative review describes the role of GI nurses in the diagnosis and treatment of IBS. Since this is a narrative review, we will not provide data from our literature search [92]. Despite little scientific evidence in the literature, we can see that nurses, if properly trained, can play a key role in the management of IBS in patients.

IBS is a condition that results in a huge expense for health care due to high outpatient admissions, prescription medications and improper instrumental examinations [9–11]. Consequently, the workload for gastroenterologists has increased. Therefore, it is essential to have trained nurses who can work alongside the gastroenterologist in managing these patients.

In some countries such as the United States and the United Kingdom, the Advanced Nurse Practitioner (ANP) has emerged. This job role was defined in 2008 by the International Council of Nurses as “one who has acquired, through additional education, the expert knowledge base, complex decisionmaking skills and clinical competencies for expanded nursing practice, the characteristics of which are shaped by the context in which they are credentialed to practice” [93]. The ANP has considerable autonomy and is involved in patient care, leadership, education, research, guideline development, and administrative tasks [94]. ANPs can occur in either generalist areas such as general medicine or in specialty areas [95].

In patient management they can:

- Conduct a comprehensive and systematic patient history, physical examination, and evaluation
- Screen patients for early signs of disease and risk factors.
- Make diagnostic decisions based on interpretation of clinical and other findings, such as laboratory results and x-rays.
- Prescribe treatment, including medications, based on a sound knowledge of pharmacology.
- Develop a combined and individualized medical and nursing care plan.
- Perform, where appropriate, simple invasive and non-invasive diagnostic and therapeutic procedures

In the field of gastroenterology, the application of the ANP figure in clinical practice is being tested. Previous studies, for example, supported the role of the “nurse endoscopists” trained to perform flexible sigmoidoscopy to face the increasing demand for colorectal cancer screening [96]. However, there are no standard guidelines as to the number of cases which the nurse endoscopist should perform before being considered competent, only a few studies [97–99].

To date, therefore, there is a lack of regulation on the training pathway of ANPs as well as regulation from the insurance point of view, although progress is being made in this regard [100].

In Italy there is no provision for this professional role. Our goal is to begin a deep reflection of the need to improve standards for education, certification, and regulation for specialist nurses to enable a better management of IBS through their support. This contribution of GI nurses could lead to an improvement in the patient’s quality of life, better management of symptoms, and less need to access hospital facilities resulting in reduced costs for public health.

## 8. Conclusions

In conclusion, considering that IBS patients need a multidimensional approach, nurses could become key players in the collaborative process of patient care by helping to guide them in choosing the most appropriate treatment and in monitoring them over time.

## Figures and Tables

**Table 1:** Summary of selected studies on the role of the nurse in the management of IBS.

Article	Design	Objectives	Results
[79]	Original Article	Determine nurses’ knowledge of IBS including diagnosis, etiology, disease impact, and management	Only 13 % were aware of the diagnostic criteria for IBS.
[80]	Systematic Review	Investigate the efficacy and overall effect of self-management interventions for patients with IBS	There is robust evidence supporting self-management interventions for improving short-term symptom management and improving quality of life, whereas longer-term outcomes are variable.
[81]	Review	We suggest ways to advance research methods and practical applications of self-management as steps in its future development and implementation.	We suggest ways to advance research methods and practical applications of self-management as steps in its future development and implementation.
[82]	Randomized Controlled Trial	Compare the effects of long multidisciplinary group education with a short nurse-based group education in IBS patients.	However, positive effects on symptoms, knowledge, quality of life, and satisfaction with the intervention were found in both the short and the long version.
[83]	Randomized Controlled Trial	Examine the effect of a nurse-led self-management program on pain, symptoms, and quality of life among young adults with IBS	Increased self-efficacy mediated the intervention effect of the Nurse-Led Online Modules group on reducing pain interference and improving quality of life
[85]	Review	Discuss ways to optimally use the FODMAP diet in practice in a wide range of cultures, directed at gastroenterologists from a dietitian’s perspective.	The diet can be delivered by other health professionals such as the gastroenterologist or nurse, but training on how to do so successfully would be needed.
[90]	Original Article	Quantified health-related quality of life in a group of IBS patients and measures changes following a treatment programme of nurse-led gut-directed hypnotherapy.	Gut-directed hypnotherapy has a very positive impact on health-related quality of life with improvements in psychological well-being and physical symptoms.
[91]	Observational Study	Evaluate the efficacy of Nurse practitioner-delivered cognitive-behavioral treatment in IBS patients	Treatment satisfaction was high. There were improvements in clinical outcomes across treatment with large effects for IBS-symptom severity 95 % CI = 0.5, 1.5) and IBS quality of life 95 % CI = 0.4, 1.2).

## Data Availability

Not applicable.
